# PTM-PAL Pipeline: A Python Web Platform of Integrative Scoring System for Assessing the Functional Impact of Protein S-Palmitoylation

**DOI:** 10.3390/membranes16100341

**Published:** 2026-10-14

**Authors:** Xin-Yu Bi, Jia-Ni Chen, Xiong-Fei Pan, Ling Tao

**Affiliations:** 1 Department of Nutrition and Food Hygiene, School of Public Health, Institute of Nutrition, Fudan University, Shanghai 200032, China; 2 Department of Good Clinical Practice, Medical Guarantee Center, Second Affiliated Hospital of Naval Medical University, Shanghai 200003, China; 3 Independent Researcher, Shanghai 201210, China

**Keywords:** post-translational modification, S-palmitoylation, functional-impact prioritization, membrane coupling, web service

## Abstract

S-palmitoylation is a reversible lipid modification that can alter protein localization, stability, interactions and signaling, but detection of a modified cysteine does not establish its functional importance. Post-translational Modification-Palmoylation Pipeline(PTM-PAL) is a web-accessible workflow for prioritizing candidate S-palmitoylation sites and modification configurations for functional follow-up. It integrates site-specific evolutionary context and UniProt annotation, paired wild-type and palmitoylated structural models, local protein-body solvent-accessible surface-area remodeling, and membrane-embedding descriptors. The workflow takes site metadata, full-length sequences and paired WT/PAL structural models as input and returns dimension-resolved outputs together with a within-protein score on a 0–10 scale. Single-site and dual-site configurations can be examined separately, while the underlying measurements remain available for inspection. PTM-PAL is intended to organize heterogeneous biochemical, structural and membrane-related evidence into testable priorities; the score is not a calibrated probability and does not replace biochemical or functional validation.

## 1. Introduction

S-palmitoylation is a reversible lipid modification in which the 16-carbon saturated fatty acid palmitate is covalently attached to specific cysteine residues through a thioester bond [1,2,3]. This modification is widespread across eukaryotes, from yeast to humans. It is catalyzed by palmitoyl acyltransferases (the DHHC family) [4,5,6] and can be rapidly removed by depalmitoylating enzymes (such as APT1) [7], thereby forming a dynamic regulatory cycle. By adding a hydrophobic lipid tail to proteins, S-palmitoylation regulates multiple cellular processes, including protein anchoring to specific membrane compartments [8], protein stability [9,10,11], protein–protein interactions [12,13], and signal transduction [13]. For example, in the nervous system, many synapse-associated proteins undergo this modification, which is indispensable for synaptic plasticity and neurotransmitter release [14,15]. In cancer, aberrant palmitoylation of small GTPases such as Ras can drive uncontrolled signaling [16]. Dysregulated palmitoylation has also been linked to Huntington’s disease [17], Alzheimer’s disease [18], intellectual disability [19], and schizophrenia [20]. Therefore, both palmitoylation and the enzymes that regulate it have emerged as promising therapeutic targets.

Mass spectrometry (MS)-based proteomic approaches, including acyl-biotinyl exchange (ABE) [21], acyl-resin capture, and click chemistry, have enabled the identification of thousands of candidate palmitoylated proteins at the cellular or tissue level. However, datasets generated by different laboratories or experimental methods often show substantial variability and may contain false positives or nonspecific background modifications. Consequently, functionally important palmitoylated proteins involved in specific biological processes can be obscured by large-scale datasets, making efficient prioritization and experimental validation challenging.

Computational tools including CSS-Palm, CKSAAP-Palm and GPS-Palm prioritize candidate modification sites from sequences and related features [22,23,24]. Functional interpretation requires additional information about the residue and its structural environment. Conservation can identify constrained positions, whereas local structural exposure and membrane association describe the distinct consequences of adding a lipid chain [25,26,27]. These quantities can inform experimental prioritization, although none alone measures the functional effect of palmitoylation.

PTM-PAL analyzes paired wild-type (WT) and palmitoylated (PAL) structures together with full-length sequences and site metadata. It combines biological context, local structural remodeling and membrane-positioning calculations into three dimension-specific scores. The resulting total is intended to compare cysteines or modification configurations within the same protein. We examined a literature-curated panel of membrane-associated proteins and related the component scores to site-specific experimental findings. PTM-PAL integrates biological, structural, and membrane-related evidence to prioritize candidate S-palmitoylation sites within individual proteins, rather than to predict whether a given site is modified under specific biological conditions or as a cross-protein score of effect size.

## 2. Materials and Methods

### 2.1. Web Application and User Workflow

**Figure 1:**
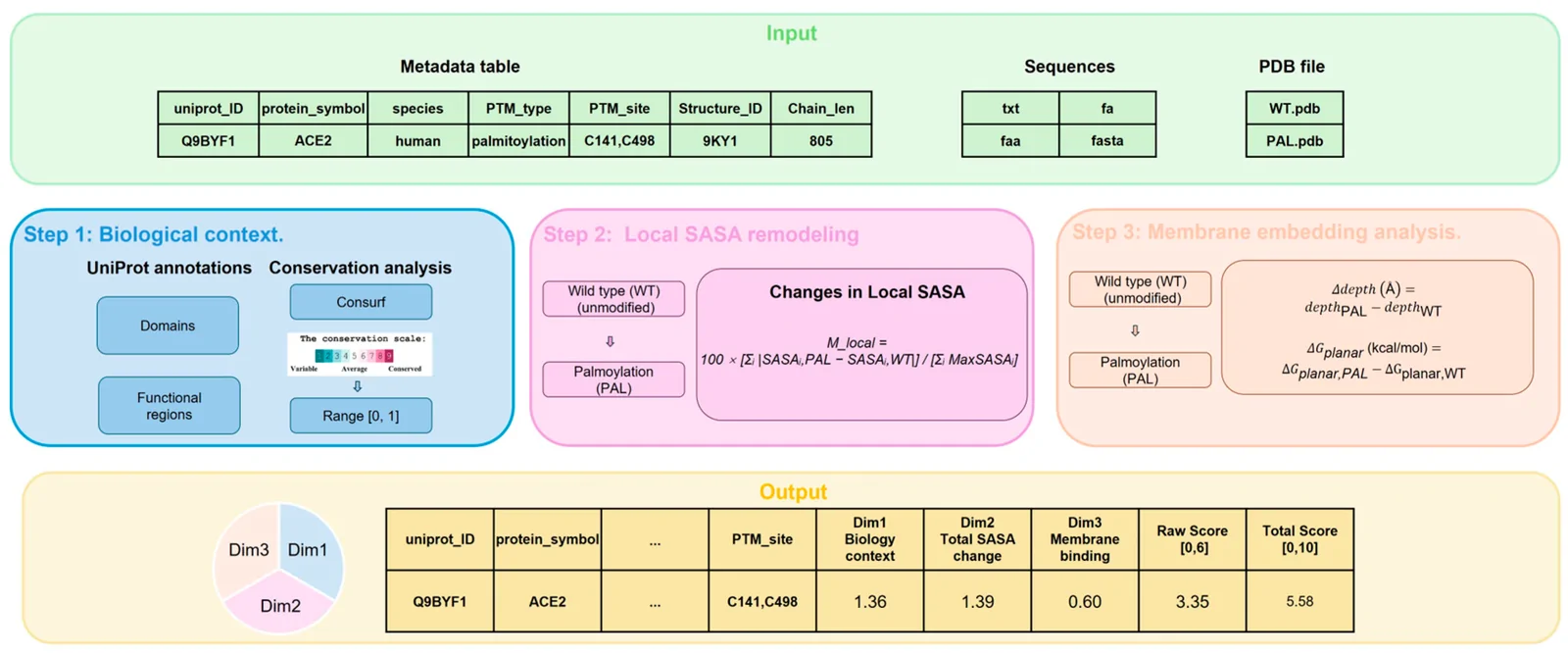
PTM-PAL workflow. The workflow diagram summarizes the input metadata, sequences and paired structures, followed by biological-context, local SASA-remodeling and membrane-positioning analyses and the dimension-resolved output.

**Figure 2:**
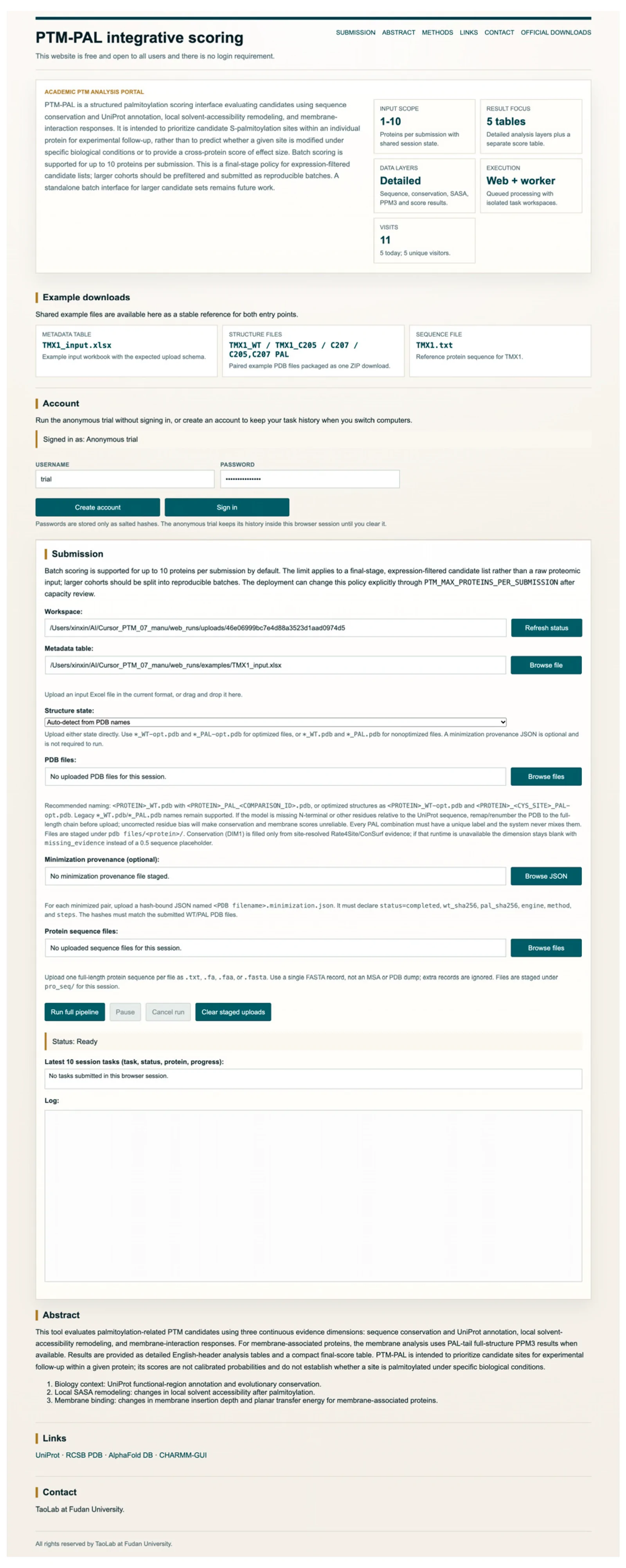
The PTM-PAL pipeline web service.

The Python 3.11.15 workflow takes an Excel metadata table, full-length sequences and paired WT/PAL PDB files as input. It integrates UniProt annotation, ConSurf conservation, SASA calculations and PPM 3.0 analysis, with stepwise logs and tabular exports. Detailed module measurements and a separate final-score workbook retain the input identifiers, dimension scores, raw applicable-dimension sum and the rescaled 0–10 score. The web service is available at https://pphome.chat/PTM-Pipeline (accessed on 7 October 2026). The web layer uses Python’s standard-library http.server with ThreadingHTTPServer and BaseHTTPRequestHandler. Each submission receives a session-isolated task workspace, and a worker polls the task store and returns the result package. The workflow is summarized in Figure 1, and the web interface is shown in Figure 2. A comparison of representative computational approaches relevant to protein S-palmitoylation is provided in Additional File S1, Table S1.

### 2.2. Structure Preparation

Structural inputs were obtained from the RCSB Protein Data Bank and AlphaFold Protein Structure Database [28,29]. WT and PAL models were prepared using the CHARMM-GUI PDB Reader & Manipulator [30], with explicit palmitoyl groups attached to the designated cysteines in PAL models. Amber ff19SB was used for the protein, and the palmitoyl group and the cysteine–palmitoyl junction were represented with GAFF2 and AM1-BCC-derived parameters. Topology and coordinate files were generated in LEaP, and each system was solvated in a rectangular TIP3P water box with a 10 Å buffer and neutralized with Na^+^ and Cl^−^ ions. Each WT/PAL pair was then subjected to three-stage energy minimization: solvent, ions and hydrogen atoms were relaxed while solute heavy atoms were restrained; backbone atoms were restrained while side chains and solvent were relaxed; and the complete system was minimized without restraints. No heating, equilibration or production molecular-dynamics simulation was performed. Water and ions were removed after minimization, and the minimized solute coordinates were exported for paired SASA and membrane analyses. Residue numbering was checked against the full-length sequence to identify target sites; modeled completion of missing residues was distinguished from coverage in the original experimental structure. Individual and dual-site PAL configurations were analyzed separately against the corresponding WT input.

### 2.3. Computational Modules and Integrated Scoring

Each task workspace is structured into three directories: “input”, “data_output”, and “pipeline_step_logs”. The pipeline is implemented in Python 3 using pandas, openpyxl, requests, tqdm, and Biopython. UTF-8 encoding is enforced throughout all subprocesses to ensure reproducible and platform-independent file processing.

### 2.4. Step 1: Biological Context

#### 2.4.1. UniProt Functional Annotation

The UniProt REST API supplied sequence identifiers, protein sequences and residue-overlapping feature annotations [31]. The annotation subscore followed a highest-tier rule: ACT_SITE, BINDING or SITE contributed 1; DOMAIN, REGION, MOTIF, COMPBIAS, DNA_BIND, ZN_FING, TRANSMEM, INTRAMEM or TOPO_DOM contributed 0.5; absence of a qualifying feature contributed 0. Overlapping features were not added. LIPID, MOD_RES, DISULFID and CROSSLNK features were retained for provenance and quality control but did not automatically increase the score.

#### 2.4.2. Evolutionary Conservation

Full-length query sequences were analyzed in Sequence mode using the ConSurf Colab workflow [25]. The recorded search settings used MMseqs2/UniRef, an E-value cutoff of 1 × 10^−4^, 35–95% sequence identity, a minimum query-length fraction of 0.60 and a maximum final alignment size of 100 sequences. MAFFT used the local-pair strategy with 1000 iterative refinements. ProtTest selected the protein-specific substitution model under AICc, and Rate4Site used empirical-Bayesian estimation [25]. For conversion to the continuous 0–1 component, we followed the normalization scheme used by Schmitz et al. [32]: negative Rate4Site values were mapped to 0.5–1, positive values to 0–0.5, and zero to 0.5, using separate min–max transformations on each side. Higher transformed values denote greater conservation. Dim1 was the sum of this conservation component and the UniProt annotation subscore, with a range of 0–2. Per-protein execution records, rather than the nominal alignment cap, determine the actual sequence count and selected model.

### 2.5. Step 2: Local Structural-Exposure Change (Dim2)

SASA was calculated on paired minimized structures with Biopython’s Shrake–Rupley algorithm [33] using 1000 sphere points. The local environment was defined as the union of complete protein residues containing at least one common protein heavy atom within 8 Å of the WT cysteine sulfur atom(s). For each selected residue i, SASA was summed over the shared protein heavy-atom keys present in both WT and PAL structures and normalized by its residue-specific maximum accessible surface area. Palmitoyl-tail atoms, solvents, ligands and explicit hydrogens were excluded from this protein-body metric. The PAL calculation nevertheless used the complete minimized PAL structure when evaluating the shared protein atoms, so shielding of protein atoms by the attached tail remained represented. The local remodeling magnitude was calculated as $M_{local}=100\times \frac{\Sigma_{i}\left|{SASA}_{i,PAL}-SASA_{i,WT}\right|}{\Sigma_{i}{MaxSASA}_{i}}$. For dual-site configurations, overlapping local neighborhoods were merged before the sum. The signed net change was retained separately to indicate net exposure or burial but was not used for Dim2. Dim2 was calculated as min(2, max(0, M_local/2)); thus, local remodeling values from 0 to 4% map linearly to 0 to 2 points. This metric describes a difference between modeled conformations; it does not measure modification abundance or an experimental change in accessibility.

### 2.6. Step 3: Membrane-Coupling Analysis (Dim3)

PPM 3.0 was used in its local full-structure implementation to compare the membrane positioning of WT and PAL models [27]. The full_structure mode retained the explicit PAL tail. For each protein, the WT and corresponding PAL structure were analyzed with the same membrane code, N-terminal topology, one-membrane setting, heteroatom setting and curvature setting; these parameters were assigned at the protein level and were not reselected for individual cysteine sites. Changes were defined as PAL minus WT. The depth subscore was min(1, max(0, Δdepth/1 Å)); the energy subscore was min(1, max(0, −ΔΔGplanar/1 kcal mol^−1^)). Dim3 was their sum, ranging from 0 to 2. Positive depth changes and more-negative planar transfer-energy changes therefore contribute continuously from zero. Tilt and curvature did not enter the score. Alternative in/out runs for the same protein-site configuration were not counted as independent observations.

### 2.7. Step 4: Evidence Integration and Functional Impact Scoring

For transmembrane and peripheral membrane proteins, the raw score was Dim1 + Dim2 + Dim3, with a maximum of 6; the final score was 10 × raw score/6. For soluble proteins, only Dim1 and Dim2 applied, with a maximum raw score of 4 and final score of 10 × raw score/4. An unavailable applicable dimension produced a missing total rather than an imputed zero. The continuous scoring rules are summarized in Additional File S1, Table S2, and the complete ConSurf parameter record is provided in Additional File S1, Table S3.

Sensitivity A assigned Dim1 + 2 × Dim2 + 2 × Dim3 to membrane proteins and Dim1 + 4 × Dim2 to soluble proteins. Sensitivity B omitted Dim3 only for peripheral membrane proteins, using 2.5 × (Dim1 + Dim2); other protein classes retained the main score. These analyses test dependence on the weighting scheme and are not independent validations. The scaling caps are modeling choices, rather than experimentally established thresholds of functional importance.

The evaluation panel was assembled from site-focused palmitoylation literature with biochemical and functional evidence. The initial input set contained 16 proteins and is documented in Additional File S1. IFNGR1 and PLD2 were assessed but excluded from the retained scoring panel because of structure-confidence concerns: IFNGR1 had a global AlphaFold mean pLDDT of 66.01, whereas PLD2 had target-site mean pLDDT values of 44.56–46.81. SQSTM1 was retained as a biologically motivated low-confidence structural exception. Structural coverage and local confidence were therefore reported separately rather than represented as a uniform exclusion filter. The selected proteins provided comparisons across different experimental contexts. Literature records involving more than one cysteine were classified separately from direct site-level orderings, and joint-site evidence was not treated as an independent single-site ranking test. Comparisons were restricted to matched relationships within a protein and, where possible, within a study. Literature-unreported candidates were not treated as experimentally confirmed negatives. The descriptive concordance analysis was retrospective, and comparisons sharing a protein were not assumed to be statistically independent.

## 3. Results

### 3.1. Overall Performance in a Literature-Curated Membrane Protein Panel

**Figure 3:**
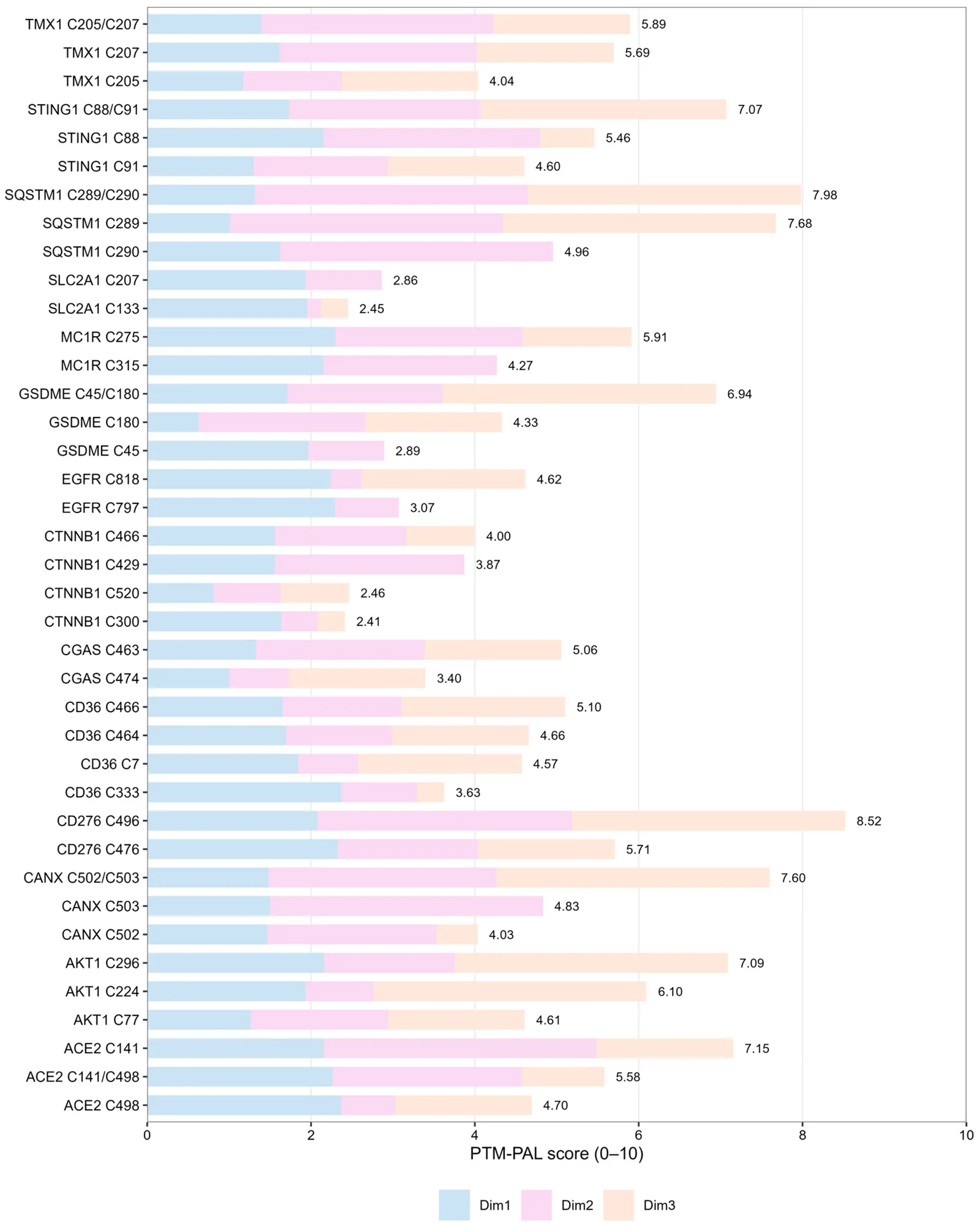
Dimension-resolved scores for all 39 configurations. Each stacked segment represents (10/6) × the dimension score, so segment lengths sum to the total on the 0–10 scale. Protein-site configurations appear once after orientation deduplication. Ordering is within-protein and does not imply comparable functional importance between proteins. Bars are deterministic model outputs, not replicate means.

**Figure 4:**
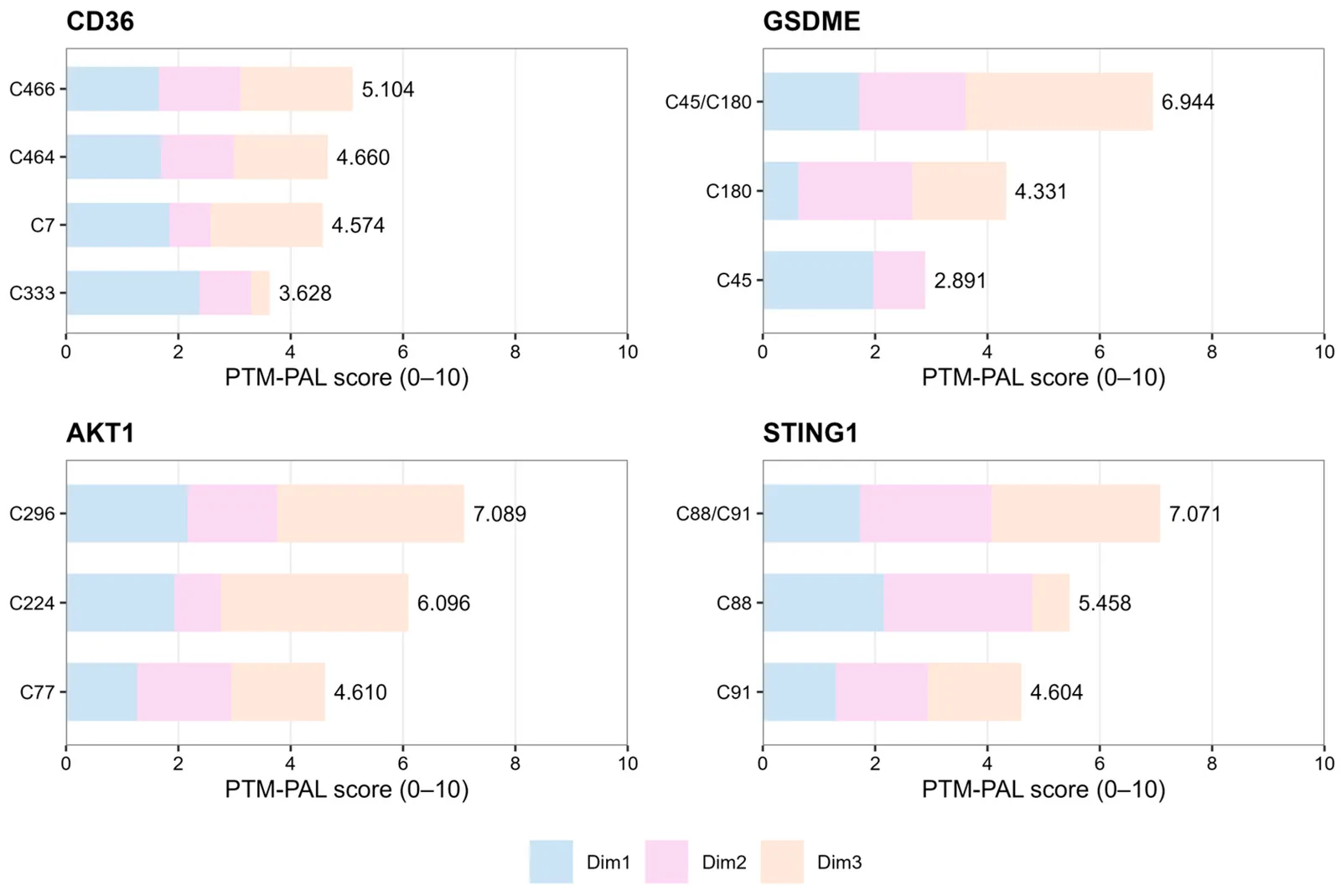
Four case studies with dimension-resolved scores. CD36, GSDME, AKT1 and STING1. Colors and scaling match Figure 3. C333 in CD36 and C296 in AKT1 are literature-unresolved candidates in these comparisons. Numerical labels show model totals; no experimental effect sizes are depicted.

The retained panel contained 39 distinct protein-site configurations across 14 proteins: 33 single-site and six dual-site configurations, from nine transmembrane and five peripheral membrane proteins. Each protein-site configuration contributed one record after deduplication of alternative PPM3 orientations. All three dimensions were applicable to this membrane-focused panel. The dimension-resolved scores varied within and between proteins (Figure 3; Additional File S1, Table S4). Four case studies examined how the same scoring framework relates to different forms of experimental evidence (Table 1; Figure 4).

The literature-compatibility analysis yielded 15 concordant relationships among 16 comparisons (93.75%; Additional File S1, Table S5). This denominator included eight comparisons involving CANX, SQSTM1, STING1 and TMX1 for which joint-site evidence supported a combined-versus-single interpretation without establishing every single-site ordering [34,35,36,37]. It therefore describes compatibility under the stated interpretation rules, not sensitivity, specificity or independent classification accuracy. ACE2 accounted for the discordant comparison: its dual-site score exceeded C498 but not C141. All 39 configurations remained in the score summary, including configurations without an adjudicable functional ranking.

Unreported candidate sites sometimes scored above experimentally studied sites. AKT1 C296 scored 7.089, exceeding C224 and C77, and CGAS C463 and MC1R C275 exceeded the reported C474 and C315 sites, respectively [38,39,40]. These results represent unresolved candidates rather than confirmed false positives, because absence from the selected literature is not negative experimental evidence. Conversely, EGFR C797 scored 3.071 despite experimental support [41], illustrating why an uncalibrated universal score cutoff would be inappropriate. Cross-protein absolute scores were not used to rank biological importance.

### 3.2. Case Study 1—Site Discrimination: CD36

CD36 C466, C464 and C7 scored 5.104, 4.660 and 4.574, respectively, all above the unreported comparator C333 (3.628). Wang et al. identified C466 as the APT1-sensitive site associated with CD36 trafficking and podocyte lipid accumulation [42]. Accordingly, C466 was the dominant ranked candidate among the three reported CD36 sites, and C7 was the lowest-scoring reported site; C333 was lower than all three reported sites. The score therefore prioritized the experimentally emphasized site. The comparison with C333 is indirect: C333 was not an experimentally established negative control. Relative to C333, C466 had a lower Dim1 score but higher Dim2 and Dim3 scores (Figure 4).

A similar balance occurred in CD276: C496 scored 8.522 versus 5.707 for C476, despite a lower Dim1 value (1.247 versus 1.395). Higher Dim2 and Dim3 values accounted for the total-score difference. The C496 palmitoylation study provides a biological reference for this comparison [43], but its AlphaFold target-site pLDDT of 69.19 limits interpretation of the modeled local geometry.

### 3.3. Case Study 2—Functional Hierarchy: GSDME

**Figure 5:**
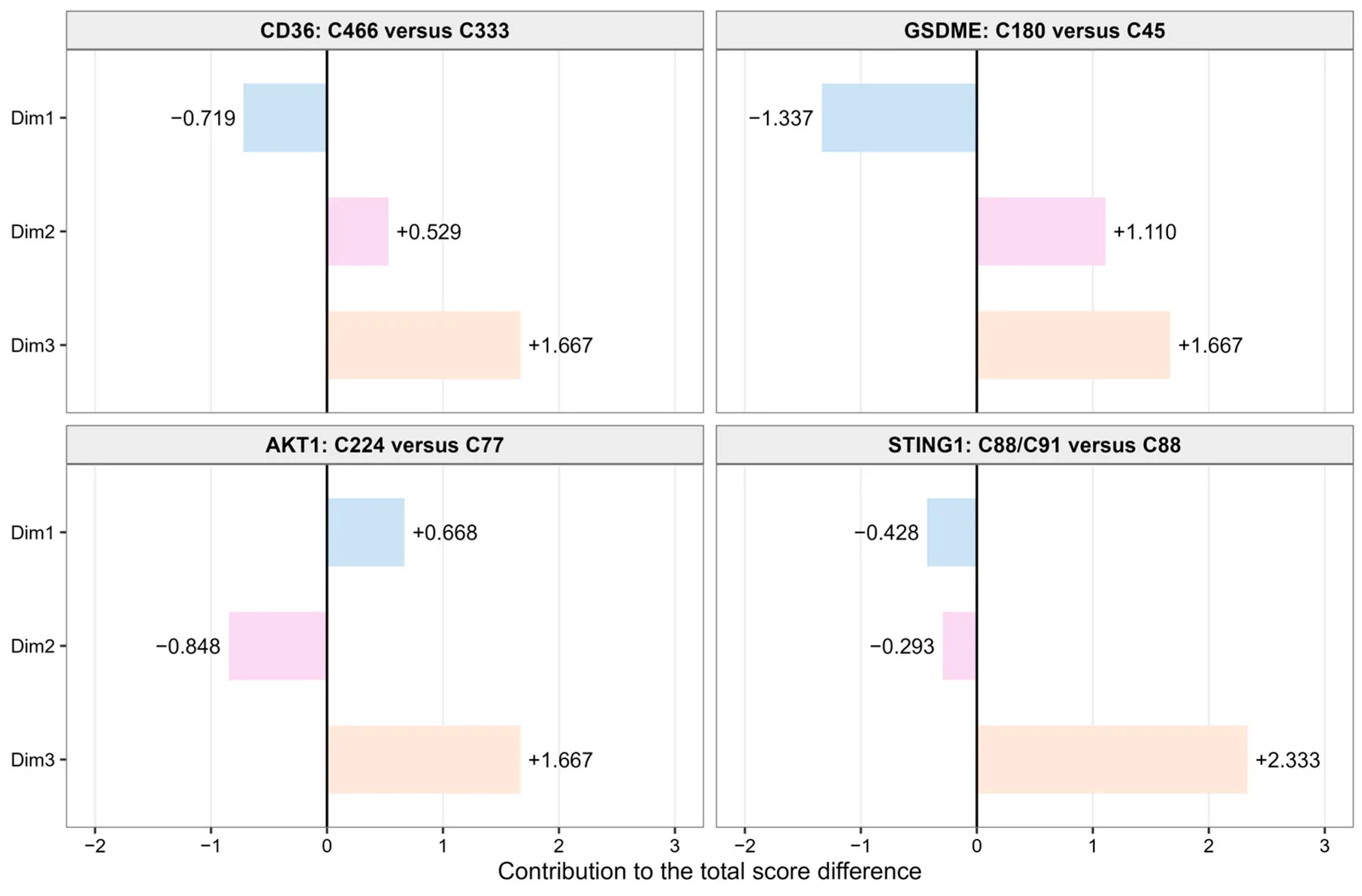
Contributions of individual dimensions to selected score differences. Each bar is (10/6) × [Dim of the first configuration − Dim of the second configuration]. Positive values favor the first configuration; negative values favor the second. The sum across dimensions equals the corresponding total-score difference. These are algebraic score contributions, not causal effects or experimental estimates.

GSDME C180 scored 4.331, above C45 at 2.891; the C45/C180 configuration scored 6.944. Teran et al. identified both sites and found stronger impairment of pore-forming activity after C180 substitution, with a context-dependent contribution from C45 (Figure 4 [44]). The score ordering was compatible with that functional distinction. The lower Dim1 score of C180 was offset by higher Dim2 and Dim3 scores (Figure 5).

Omitting Dim3 reversed the single-site ordering: the 0–10 score based on Dim1 and Dim2 alone was 4.337 for C45 and 3.997 for C180. This shows that the model’s membrane term was necessary to recover the observed ranking in this example. It does not establish that the computed energy or insertion-depth changes are the physical cause of the experimental phenotype.

### 3.4. Case Study 3—Comparison with Published In Vivo Evidence for AKT1

AKT1 C224 scored 6.096 versus 4.610 for C77. Bu et al. investigated palmitoylation using C77S/C224S knock-in cells, mutant-expression mouse models and Akt1 C224S knock-in mice [38]. These experiments connect site modification with AKT signaling and physiological phenotypes; the mouse knock-in evidence specifically concerns C224. In the computational comparison, C224 gained 0.668 total-score units from Dim1 and 1.667 from Dim3 relative to C77, while Dim2 opposed this ordering by 0.848 units (Figure 4). Removing Dim3 reversed the order, yielding 4.145 for C224 and 4.415 for C77. Published experiments provided biological support but were also used to curate the panel, so this comparison was not a held-out validation test. C224 was the dominant ranked candidate among the two experimentally compared sites. C296, the highest-scoring AKT1 candidate at 7.089, remains experimentally unresolved in this comparison.

### 3.5. Case Study 4—Structural/Membrane Interpretation: STING1

STING1 C88/C91 scored 7.071, above C88 (5.458) and C91 (4.604). Mukai et al. showed that palmitoylation of this membrane-proximal cysteine pair was required for STING signaling at the Golgi [34]. Their experiments separated signaling from trafficking: impairment of palmitoylation did not simply prevent movement from the endoplasmic reticulum to the Golgi. The combined configuration had Dim3 = 1.8, rather than the maximum of 2.0, and its advantage over C88 was attributable to Dim3 despite lower Dim1 and Dim2 values (Figure 4). This is compatible with a membrane-associated signaling role, but does not establish a biochemical cooperative effect or C88 versus C91 functional dominance.

### 3.6. Multi-Site Configurations and Weighting Sensitivity

Five of the six dual-site configurations scored above both corresponding single-site models: CANX, GSDME, SQSTM1, STING1 and TMX1. ACE2 C141/C498 scored 5.580, below C141 (7.154) but above C498 (4.696), despite literature supporting the importance of both sites [45]. For CANX and TMX1, the relevant studies connect palmitoylation to complex association or membrane-compartment targeting [35,36]; for SQSTM1, the selected study links reduced palmitoylation to impaired autophagy [37]. These findings justify examining combined configurations, but do not imply that every combined-versus-single score comparison has an experimentally measured counterpart. The score does not automatically reward the presence of a second tail. Weighting sensitivity results are provided in Additional File S1, Table S4.

### 3.7. Structural Confidence Defines the Boundary of Structure-Dependent Interpretation

Structure annotations classified 29 configurations as conforming, seven as warning and three as nonconforming. Eleven configurations used RCSB-derived models and 28 used AlphaFold-derived models. The target sites were present, but target-site presence did not guarantee complete experimental coverage or reliable local geometry. AKT1 structure 7NH5 covered 79.79% of the reference sequence; GSDME structure 9H5M covered 49.40%, whereas the analysis model covered 100%. The latter distinction reflects model completion rather than new experimental observations. SQSTM1 C289 and C290 had pLDDT values of 29.30 and 27.39, respectively, as recorded in Additional File S1. These scores were retained for transparency but were not used as reliable evidence of a specific local structural mechanism.

## 4. Discussion

### 4.1. Functional Prioritization and the Meaning of Literature Concordance

The case studies show how integrated scores can recover selected within-protein relationships while retaining unresolved or discordant configurations. The 15/16 compatibility result has a narrower meaning than overall prediction accuracy: its comparisons share proteins and include inferred joint-site relationships, and its literature was part of the panel construction. The remaining configurations are not missing observations; some lack a directly comparable functional experiment. A future external evaluation would require independent sampling, matched experimental endpoints and predefined decision rules.

A modified residue can be essential for a particular phenotype without receiving a high score across all modeled dimensions. Conversely, a high score at an unreported site does not establish that modification occurs. AKT1 C296 and the alternative candidates in CGAS and MC1R illustrate this distinction. Accordingly, the scores identify candidates for experiments within a protein; they are not calibrated probabilities or measurements of effect size across proteins. PTM-PAL is intended to prioritize candidate sites for experimental follow-up within a given protein. Its scores do not establish whether a site is palmitoylated under specific biological conditions.

### 4.2. Dim1 Relates Evolutionary Constraint to Site Context

ConSurf estimates evolutionary constraint, whereas UniProt annotates known sequence features [25,31]. These are complementary sources of context within Dim1, but neither is specific proof of palmitoylation. The site-focused literature provides a test of how such context behaves after integration. For example, the GSDME functional experiments support a stronger role for C180 than C45 [44], although C180 has a lower Dim1 score. CD36 C466 likewise has a lower Dim1 score than C333, while Wang et al. identify C466 as the relevant APT1-sensitive site [42]. Thus, the biological case for these positions comes from experimental evidence; the framework recovers their priority only when additional modeled properties are considered.

Dim1 should not be described as conservation alone, because it also includes a categorical annotation component. In AKT1, Dim1 favors C224 over C77, while Dim2 favors C77. The total ordering therefore results from the measured combination of subscores rather than a general rule that conserved sites are more important (Figure 4). Feature provenance also matters: lipid-modification annotations are not automatically rewarded, reducing the direct reuse of the palmitoylation label as a scoring input.

### 4.3. Dim2 Measures Structural Remodeling Rather than Its Functional Direction

The GSDME study provides a structural context for interpreting local exposure: C45 occupies a lipid-binding tip whose environment changes between inhibited and pore-forming states, whereas C180 lies in a membrane-engaging region [44]. This supports examining the local structure, but does not validate a particular SASA score. Dim2 measures the magnitude of local protein-body remodeling. An increase in exposure and an increase in burial can both contribute; a higher value therefore cannot be translated into greater accessibility, stronger function or enhanced membrane binding without examining the underlying geometry.

The distinction is visible in the score decomposition. Dim2 favors GSDME C180 over C45 but opposes AKT1 C224 over C77. CANX palmitoylation is implicated in its association with the ribosome–translocon complex [35], and TMX1 palmitoylation is associated with mitochondria-associated membrane targeting [36]. Such observations identify biological outcomes to investigate, rather than providing experimental confirmation that a computed SASA change measures complex affinity or targeting efficiency. Paired structure preparation, flexible regions and alternative conformations may affect Dim2; the 4% cap is a scoring convention that requires independent calibration.

### 4.4. Dim3 Relates the PAL Tail to Membrane-Associated Phenotypes

PPM 3.0 describes the energetically favorable placement of a structure in an implicit membrane environment [27]. Retaining the PAL tail allows the paired calculation to represent the added hydrophobic group. The relevance of membrane effects is supported by the site-specific biology: AKT1 palmitoylation is associated with membrane localization and signaling [38], CD36 depalmitoylation changes trafficking [42], and STING palmitoylation supports signaling at the Golgi [34]. These are distinct phenotypes, so a favorable modeled depth or transfer-energy change should not be equated with a single universal mechanism.

For AKT1 and GSDME, the removal of Dim3 reverses the selected single-site ordering. For STING1, Dim3 accounts for the dual-site score advantage despite opposing changes in the other two dimensions. These results identify exactly where the membrane assumption affects prioritization. They do not prove that deeper insertion causes activation, and the STING experiments caution against treating loss of signaling as loss of Golgi trafficking [34]. The 1 Å and 1 kcal mol^−1^ caps provide bounded scaling; the cited biological studies do not establish those numerical boundaries.

### 4.5. Multi-Site Evidence and Structural Uncertainty

Combined-site models permit comparison with experiments that perturb more than one cysteine, but a higher combined score is not, by itself, evidence of a cooperative effect. Evidence of joint dependence, an experimentally resolved hierarchy and a quantified interaction between sites are different levels of support. This distinction is especially relevant to CANX, TMX1 and SQSTM1 [35,36,37]. SQSTM1 was retained because of biological interest despite very low local pLDDT; its high scores cannot resolve the underlying structural uncertainty. Restricting the compatibility analysis to configurations outside SQSTM1 yields 13/14 concordant relationships, which remains a descriptive, dependent comparison rather than an independent accuracy estimate.

### 4.6. Limitations and Future Development

PTM-PAL was developed to integrate site annotation, evolutionary conservation, local structural remodeling, and membrane-positioning measurements to prioritize candidate S-palmitoylation sites within a protein for experimental follow-up. The present results, derived from a retrospective and literature-curated evaluation of membrane-associated proteins, support the utility of PTM-PAL for generating within-protein prioritization hypotheses and provide a basis for future validation across diverse datasets and protein contexts. The utility of PTM-PAL will depend not only on the scoring framework but also on the reproducibility, reliability, and scalability of its implementation.

PTM-PAL already displays dimension-resolved scores and visual comparisons of candidate sites within each protein. Future work will improve the documentation of paired WT/PAL structure preparation and the consistency of structural-quality checks. Processing time and task-failure rates will be measured across representative submissions before assessing caching and parallel execution. A standalone batch interface could facilitate the analysis of larger candidate sets. The interface could also be expanded to visualize structural-quality measures and results from each analytical step alongside the existing score plots.

Finally, broader application to soluble proteins and other protein classes will require further refinement and independent validation of the relevant scoring rules. By coupling transparent, dimension-resolved outputs with reproducible and accessible software delivery, PTM-PAL may help advance palmitoylation research from large-scale site discovery toward hypothesis-driven and experimentally testable prioritization.

## 5. Conclusions

PTM-PAL combines evolutionary and annotation context, local structural remodeling and membrane-coupling calculations for the within-protein prioritization of candidate palmitoylation configurations. The dimension-resolved analysis explains selected literature-compatible rankings and exposes cases in which membrane assumptions or structural uncertainty dominate the result. The framework provides testable priorities while leaving modification status and functional effects to experimental validation.

## Figures and Tables

**Table 1:** Four case studies and the scope of their literature comparison.

Case	Literature Comparison	PTM-PAL Result	Interpretation
CD36	C466, C464 and C7 reported; C333 unreported neighbor	C466 5.104 > C464 4.660 > C7 4.574 > C333 3.628	C466 dominant among reported sites; C7 lowest reported site; C333 lower than all three reported sites.
GSDME	C180 primary; C45 context dependent	Dual 6.944 > C180 4.331 > C45 2.891	C180 was the dominant single-site candidate, consistent with the stronger reported C180 effect.
AKT1	C77/C224 experiments; C224S mouse knock-in	C224 6.096 > C77 4.610	C224 was the dominant ranked candidate among the two experimentally compared sites; literature informed panel construction.
STING1	Joint-site signaling requirement	Dual 7.071 > C88 5.458 and C91 4.604	The dual-site configuration exceeded both single sites, consistent with a joint requirement; this is not evidence of synergy.

## Data Availability

The original contributions presented in this study are included in the article/Supplementary Material. Further inquiries can be directed to the corresponding author.

## Supplementary Materials

The following supporting information can be downloaded at https://www.mdpi.com/article/10.3390/membranes16100341/s1, Table S1: Comparison of representative computational approaches relevant to protein S-palmitoylation, Table S2: Scoring rules and applicability, Table S3: ConSurf and Rate4Site parameter record. Table S4: Scores and weighting sensitivity for all configurations, Table S5: Comparison with site-specific literature evidence. References [22,23,24,34,35,36,37,38,42,44,45,46,47,48,49,50,51,52,53,54,55,56] are cited in supplementary materials.

## Abbreviations

The following abbreviations are used in this manuscript:

PAM-PAL	post-translational modification palmitoylation pipeline
ABE	acyl-biotinyl exchange
API	application programming interface
MS	mass spectrometry
PAL	palmitoylated
PDB	Protein Data Bank
PPM	Positioning of Proteins in Membranes
PTM	post-translational modification
RCSB	Research Collaboratory for Structural Bioinformatics
REST	Representational State Transfer
SASA	solvent-accessible surface area
WT	wild-type
