# Supplementary material for: PTM-PAL Pipeline: A Python Web Platform of Integrative Scoring System for Assessing the Functional Impact of Protein S-Palmitoylation
Source: Membranes (Basel). 2026 Oct 14;16(10):341. doi: 10.3390/membranes16100341 (PMC13707211; doi:10.3390/membranes16100341)
Supplement: Supplementary file 1 [file membranes-16-00341-s001.zip › membranes-4428710-supplementary.pdf]

membranes

Supplementary Materials

PTM-PAL Pipeline: A Python Web Platform of Integrative Scoring System for Assessing the Functional Impact of Protein S-palmitoylation

Xin-Yu Bi [1], Jia-Ni Chen [1,2], Xiong-Fei Pan [3] and Ling Tao [1,*]

[1] Department of Nutrition and Food Hygiene, School of Public Health, Institute of Nutrition, Fudan University, Shanghai 200032, China
[2] Department of Good Clinical Practice, Medical Guarantee Center, Second Affiliated Hospital of Naval Medical University, Shanghai 200120, China
[3] Independent Researcher, Shanghai 201210, China
* Correspondence: ling_tao@fudan.edu.cn

Table S1. Comparison of representative computational approaches relevant to protein S-palmitoylation.

Tool / year	Reference	Model characteristics															
		S-palmitoylation-specific	Multi-PTM framework	Site-level output	Site-occurrence prediction	Functional-impact assessment	Handcrafted sequence features	Evolutionary profile/conservation	Deep learning	Protein language model (PLM)	Structure-derived features	Explicit 3D structure analysis	Paired WT/PAL structures	ΔSASA analysis	Membrane topology/localization	Membrane energetics/orientation	Web/Software available
PTM-PAL (this study)	This study	✓		✓		✓		✓			✓	✓	✓	✓	✓	✓	✓
Deep-Palm (2026)	Deng et al., 2026[a][1]	✓		✓	✓		✓		✓	✓	✓	✓					
SAPPTree (2026)	Guo et al., 2026[a][2]	✓		✓	✓		✓				✓	✓					✓
TopoPalmTree (2025)	Forrester et al., 2025[3]	✓		✓	✓		✓				✓				✓		
MTPrompt-PTM (2025)	Han et al., 2025[4]		✓	✓	✓				✓	✓	✓						
PTMGPT2 (2024)	Shrestha et al., 2024[5]		✓	✓	✓				✓	✓							✓
GPS-Palm (2021)	Ning et al., 2021[6]	✓		✓	✓		✓	✓	✓		✓						✓
pCysMod (2021)	Liu et al., 2021[7]		✓	✓	✓		✓		✓								✓
RFCM-PALM (2021)	Bandyopadhyay et al., 2021[8]	✓		✓	✓		✓										
SPalmitoylC-PseAAC (2019)	Hussain et al., 2019[9]	✓		✓	✓		✓										✓
CapsNet-PTM (2019)	Wang et al., 2019[10]		✓	✓	✓				✓								
Li et al. integrated SVM (2017)	Li et al., 2017[11]	✓		✓	✓		✓	✓									
MDD-Palm (2017)	Weng et al., 2017[12]	✓		✓	✓		✓	✓			✓						✓
CKSAAP-Palm (2009)	Wang et al., 2009[13]	✓		✓	✓		✓										✓

		Model characteristics															
**Tool / year**	**Reference**	**S-palmitoylation-specific**	**Multi-PTM framework**	**Site-level output**	**Site-occurrence prediction**	**Functional-impact assessment**	**Hand-crafted sequence features**	**Evolutionary profile/conservation**	**Deep learning**	**Protein language model (PLM)**	**Structure-derived features**	**Explicit 3D structure analysis**	**Paired WT/PAL structures**	**ΔSASA analysis**	**Membrane topology/ localization**	**Membrane energetics/ orientation**	**Web/Software available**
CSS-Palm 2.0 (2008)	Ren et al., 2008[14]	✓		✓	✓		✓										✓

Notes. √ indicates features explicitly described or assessed in the cited source. Abbreviations: S-pal, S-palmitoylation; PTM, post-translational modification; PLM, protein language model; WT/PAL, paired wild-type/palmitoylated structures; ΔSASA, change in solvent-accessible surface area; 3D, three-dimensional structure.
[a]Preprint available at the time of the search.

Table S2. Scoring rules and applicability.

Dimension	Definition	Range
Dim1	Site-specific conservation (0–1) + highest-tier UniProt annotation (0, 0.5 or 1)	0–2
Dim2	min(2, max(0, local SASA remodeling magnitude % / 2))	0–2
Dim3 depth	min(1, max(0, (depthPAL − depthWT)/1 Å))	0–1
Dim3 energy	min(1, max(0, −(Gplanar,PAL − Gplanar,WT)/1 kcal mol−1))	0–1
Dim3	Depth subscore + energy subscore	0–2
Soluble total	10 × (Dim1 + Dim2)/4	0–10
Membrane total	10 × (Dim1 + Dim2 + Dim3)/6	0–10

Conservation and annotation follow Methods 2.4. Structural terms are continuous and capped; the UniProt annotation component is categorical. Missing applicable dimensions yield a missing total. Structural caps are prespecified scaling limits, not biological thresholds. Table S5 separates direct site-level orderings, combined-versus-single comparisons and joint-site compatibility comparisons.

Table S3. ConSurf and Rate4Site parameter record.

Protein	UniProt	Panel status	Homolog cap	E-value	Identity range	Query-length fraction	Alignment	Substitution model	Rate4Site
ACE2	Q9BYF1	Included in retained scoring panel	100	1×10^{-4}	35–95%	0.60	MAFFT --localpair --maxiterate 1000	WAG	Empirical-Bayesian
AKT1	P31749	Included in retained scoring panel	100	1×10^{-4}	35–95%	0.60	MAFFT --localpair --maxiterate 1000	WAG	Empirical-Bayesian
CANX	P27824	Included in retained scoring panel	100	1×10^{-4}	35–95%	0.60	MAFFT --localpair --maxiterate 1000	WAG	Empirical-Bayesian
CD276	Q5ZPR3	Included in retained scoring panel	100	1×10^{-4}	35–95%	0.60	MAFFT --localpair --maxiterate 1000	JTT	Empirical-Bayesian
CD36	P16671	Included in retained scoring panel	100	1×10^{-4}	35–95%	0.60	MAFFT --localpair --maxiterate 1000	WAG	Empirical-Bayesian
CGAS	Q8N884	Included in retained scoring panel	100	1×10^{-4}	35–95%	0.60	MAFFT --localpair --maxiterate 1000	JTT	Empirical-Bayesian
CTNNB1	P35222	Included in retained scoring panel	100	1×10^{-4}	35–95%	0.60	MAFFT --localpair --maxiterate 1000	JTT	Empirical-Bayesian

Protein	UniProt	Panel status	Homolog cap	E-value	Identity range	Query-length fraction	Alignment	Substitution model	Rate4Site
EGFR	P00533	Included in retained scoring panel	100	1×10^{-4}	35–95%	0.60	MAFFT --localpair --maxiterate 1000	JTT	Empirical-Bayesian
GSDME	O60443	Included in retained scoring panel	100	1×10^{-4}	35–95%	0.60	MAFFT --localpair --maxiterate 1000	JTT	Empirical-Bayesian
IFNGR1	P15260	Excluded after structure-quality review	100	1×10^{-4}	35–95%	0.60	MAFFT --localpair --maxiterate 1000	Not retained	Not retained
MC1R	Q01726	Included in retained scoring panel	100	1×10^{-4}	35–95%	0.60	MAFFT --localpair --maxiterate 1000	JTT	Empirical-Bayesian
PLD2	O14939	Excluded after structure-quality review	100	1×10^{-4}	35–95%	0.60	MAFFT --localpair --maxiterate 1000	Not retained	Not retained
SLC2A1	P11166	Included in retained scoring panel	100	1×10^{-4}	35–95%	0.60	MAFFT --localpair --maxiterate 1000	LG	Empirical-Bayesian
SQSTM1	Q13501	Included in retained scoring panel	100	1×10^{-4}	35–95%	0.60	MAFFT --localpair --maxiterate 1000	JTT	Empirical-Bayesian
STING1	Q86WV6	Included in retained scoring panel	100	1×10^{-4}	35–95%	0.60	MAFFT --localpair --maxiterate 1000	JTT	Empirical-Bayesian
TMX1	Q9H3N1	Included in retained scoring panel	100	1×10^{-4}	35–95%	0.60	MAFFT --localpair --maxiterate 1000	WAG	Empirical-Bayesian

The final panel used the protein-specific substitution models selected by ProtTest under AICc: WAG for ACE2, AKT1, CANX, CD36 and TMX1; JTT for CD276, CGAS, CTNNB1, EGFR, GSDME, MC1R, SQSTM1 and STING1; and LG for SLC2A1. IFNGR1 and PLD2 are retained here as excluded inputs and did not contribute to final scores.

Table S4. Scores and weighting sensitivity for all configurations.

Protein	Site	Dim1	Dim2	Dim3	Total	Sens. A	Sens. B
ACE2	C141	1.293	2.000	1.000	7.154	7.293	7.154
ACE2	C141,C498	1.357	1.392	0.600	5.580	5.340	5.580
ACE2	C498	1.421	0.397	1.000	4.696	4.215	4.696
AKT1	C77	0.758	1.008	1.000	4.610	4.774	4.415
AKT1	C224	1.159	0.499	2.000	6.096	6.157	4.145
AKT1	C296	1.297	0.956	2.000	7.089	7.210	5.634
CD276	C476	1.395	1.029	1.000	5.707	5.453	5.707
CD276	C496	1.247	1.866	2.000	8.522	8.979	8.522
CD36	C7	1.104	0.440	1.200	4.574	4.385	4.574
CD36	C333	1.423	0.554	0.200	3.628	2.930	3.628
CD36	C464	1.016	0.780	1.000	4.660	4.576	4.660
CD36	C466	0.991	0.871	1.200	5.104	5.134	5.104
CGAS	C463	0.796	1.238	1.000	5.056	5.272	5.084
CGAS	C474	0.602	0.436	1.000	3.396	3.474	2.594
CTNNB1	C300	0.982	0.267	0.200	2.414	1.915	3.121
CTNNB1	C429	0.936	1.387	0.000	3.871	3.710	5.807
CTNNB1	C466	0.935	0.965	0.500	4.000	3.865	4.750
CTNNB1	C520	0.485	0.494	0.500	2.465	2.473	2.447
MC1R	C275	1.377	1.371	0.800	5.914	5.719	5.914
MC1R	C315	1.293	1.269	0.000	4.270	3.831	4.270
SLC2A1	C133	1.172	0.099	0.200	2.452	1.770	2.452
SLC2A1	C207	1.161	0.558	0.000	2.864	2.276	2.864
CANX	C502	0.880	1.241	0.300	4.035	3.962	4.035
CANX	C502,C503	0.890	1.668	2.000	7.597	8.226	7.597
CANX	C503	0.900	2.000	0.000	4.834	4.900	4.834
EGFR	C797	1.375	0.468	0.000	3.071	2.310	3.071
EGFR	C818	1.344	0.226	1.200	4.618	4.197	4.618
GSDME	C180	0.377	1.222	1.000	4.331	4.820	3.997
GSDME	C45	1.179	0.556	0.000	2.891	2.290	4.337
GSDME	C45,C180	1.028	1.138	2.000	6.944	7.305	5.416
SQSTM1	C289	0.606	2.000	2.000	7.677	8.606	6.515
SQSTM1	C289,C290	0.790	2.000	2.000	7.983	8.790	6.974
SQSTM1	C290	0.973	2.000	0.000	4.956	4.973	7.433
STING1	C88	1.294	1.581	0.400	5.458	5.256	5.458
STING1	C88,C91	1.037	1.405	1.800	7.071	7.448	7.071
STING1	C91	0.780	0.983	1.000	4.604	4.745	4.604
TMX1	C205	0.703	0.721	1.000	4.040	4.145	4.040
TMX1	C205,C207	0.836	1.699	1.000	5.893	6.235	5.893
TMX1	C207	0.970	1.446	1.000	5.693	5.862	5.693

All configurations are membrane associated. Main total = (10/6) × (Dim1 + Dim2 + Dim3). Sensitivity A = Dim1 + 2 × Dim2 + 2 × Dim3. Sensitivity B excludes Dim3 for peripheral proteins only.

Table S5. Comparison with site-specific literature evidence.

Protein	Literature-supported comparison	Evidence class	Score difference	Agreement
ACE2	C141/C498 > C141 [15]	Combined-versus-single comparison	−1.574	No
ACE2	C141/C498 > C498 [15]	Combined-versus-single comparison	+0.884	Yes
AKT1	C224 > C77 [16]	Direct site-level ordering	+1.486	Yes

Protein	Literature-supported comparison	Evidence class	Score difference	Agreement
CD36	C466 > C7 [17]	Direct site-level ordering	+0.531	Yes
CD36	C466 > C464 [17]	Direct site-level ordering	+0.444	Yes
GSDME	C45/C180 > C45 [18]	Combined-versus-single comparison	+4.053	Yes
GSDME	C45/C180 > C180 [18]	Combined-versus-single comparison	+2.613	Yes
GSDME	C180 > C45 [18]	Direct site-level ordering	+1.440	Yes
CANX	C502/C503 > C502 [19]	Joint-site compatibility	+3.562	Yes
CANX	C502/C503 > C503 [19]	Joint-site compatibility	+2.764	Yes
SQSTM1	C289/C290 > C289 [20]	Joint-site compatibility	+0.306	Yes
SQSTM1	C289/C290 > C290 [20]	Joint-site compatibility	+3.027	Yes
STING1	C88/C91 > C88 [21]	Joint-site compatibility	+1.612	Yes
STING1	C88/C91 > C91 [21]	Joint-site compatibility	+2.466	Yes
TMX1	C205/C207 > C205 [22]	Joint-site compatibility	+1.853	Yes
TMX1	C205/C207 > C207 [22]	Joint-site compatibility	+0.199	Yes

The 16 comparisons in Table S5 are classified as direct site-level orderings, combined-versus-single comparisons or joint-site compatibility comparisons. The eight joint-site comparisons were interpreted as compatibility with the reported multi-site relationship; they were not treated as independent single-site ranking tests. Comparisons involving literature-unreported neighbouring sites and the exploratory EGFR score cutoff were excluded from this 16-comparison tally. Because several comparisons came from the same protein, the proportion is descriptive and is not an estimate of prediction accuracy.

References

1. Deng, M.; Huang, J.; Wang, H.; Fu, S.; Wang, W.; Li, L.; Kang, Y.-J.; Xu, B. Deep-Palm: An Integrated Deep Learning Framework for Structure-Aware Pre-diction of Protein S-Palmitoylation. *bioRxiv* **2026**, https://doi.org/10.64898/2026.03.05.709753.
2. Guo, A.S.; Luong, V.S.; Petropavlovskiy, A.A.; Dang, A.; Doxey, A.C.; Sanders, S.S.; Martin, D.D.O. SAPPTree: Identification of an S-Acylation Motif Drives a Novel S-Acylation Prediction Program. *bioRxiv* **2026,** https://doi.org/10.64898/2026.06.30.735287.
3. Forrester, M.T.; Egol, J.R.; Ozbay, S.; Waddell, F.D.; Singh, R.; Tata, P.R. Topology-driven discovery of transmembrane protein S-palmitoylation. *J. Biol. Chem.* **2025**, *301*, 108259, https://doi.org/10.1016/j.jbc.2025.108259.
4. Han, Y.; He, F.; Shao, Q.; Wang, D.; Xu, D. MTPrompt-PTM: A Multi-Task Method for Post-Translational Modification Prediction Using Prompt Tuning on a Structure-Aware Protein Language Model. *Biomolecules* **2025**, *15*, 843, https://doi.org/10.3390/biom15060843.
5. Shrestha, P.; Kandel, J.; Tayara, H.; Chong, K.T. Post-translational modification prediction via prompt-based fine-tuning of a GPT-2 model. *Nat. Commun.* **2024**, *15*, 1–13, https://doi.org/10.1038/s41467-024-51071-9.
6. Ning, W.; Jiang, P.; Guo, Y.; Wang, C.; Tan, X.; Zhang, W.; Peng, D.; Xue, Y. GPS-Palm: a deep learning-based graphic presentation system for the prediction of*S*-palmitoylation sites in proteins. *Briefings Bioinform.* **2020**, *22*, 1836–1847, https://doi.org/10.1093/bib/bbaa038.

7. Li, S.; Yu, K.; Wu, G.; Zhang, Q.; Wang, P.; Zheng, J.; Liu, Z.-X.; Wang, J.; Gao, X.; Cheng, H. pCysMod: Prediction of Multiple Cysteine Modifications Based on Deep Learning Framework. *Front. Cell Dev. Biol.* **2021**, *9*, https://doi.org/10.3389/fcell.2021.617366.
8. Bandyopadhyay, S.S.; Halder, A.K.; Zaręba-Kozioł, M.; Bartkowiak-Kaczmarek, A.; Dutta, A.; Chatterjee, P.; Nasipuri, M.; Wójtowicz, T.; Wlodarczyk, J.; Basu, S. RFCM-PALM: In-Silico Prediction of S-Palmitoylation Sites in the Synaptic Proteins for Male/Female Mouse Data. *Int. J. Mol. Sci.* **2021**, *22*, 9901, https://doi.org/10.3390/ijms22189901.
9. Hussain, W.; Khan, Y.D.; Rasool, N.; Khan, S.A.; Chou, K.-C. SPalmitoylC-PseAAC: A sequence-based model developed via Chou's 5-steps rule and general PseAAC for identifying S-palmitoylation sites in proteins. *Anal. Biochem.* **2019**, *568*, 14–23, https://doi.org/10.1016/j.ab.2018.12.019.
10. Wang, D.; Liang, Y.; Xu, D. Capsule network for protein post-translational modification site prediction. *Bioinformatics* **2018**, *35*, 2386–2394, https://doi.org/10.1093/bioinformatics/bty977.
11. Li, L.; Luo, Q.; Xiao, W.; Li, J.; Zhou, S.; Li, Y.; Zheng, X.; Yang, H. A machine-learning approach for predicting palmitoylation sites from integrated sequence-based features. *J. Bioinform. Comput. Biol.* **2017**, *15*, 1650025–18, https://doi.org/10.1142/s0219720016500256.
12. Weng, S.-L.; Kao, H.-J.; Huang, C.-H.; Lee, T.-Y. MDD-Palm: Identification of protein S-palmitoylation sites with substrate motifs based on maximal dependence decomposition. *PLOS ONE* **2017**, *12*, e0179529–e0179529, https://doi.org/10.1371/journal.pone.0179529.
13. Wang, X.-B.; Wu, L.-Y.; Wang, Y.-C.; Deng, N.-Y. Prediction of palmitoylation sites using the composition of k-spaced amino acid pairs. *Protein Eng. Des. Sel.* **2009**, *22*, 707–712, https://doi.org/10.1093/protein/gzp055.
14. Ren, J.; Wen, L.; Gao, X.; Jin, C.; Xue, Y.; Yao, X. CSS-Palm 2.0: an updated software for palmitoylation sites prediction. *Protein Eng. Des. Sel.* **2008**, *21*, 639–644, https://doi.org/10.1093/protein/gzn039.
15. Xie, F.; Su, P.; Pan, T.; Zhou, X.; Li, H.; Huang, H.; Wang, A.; Wang, F.; Huang, J.; Yan, H.; et al. Engineering Extracellular Vesicles Enriched with Palmitoylated ACE2 as COVID-19 Therapy. *Adv. Mater.* **2021**, *33*, 2103471, https://doi.org/10.1002/adma.202103471.
16. Bu, L.; Zhang, Z.; Chen, J.; Fan, Y.; Guo, J.; Su, Y.; Wang, H.; Zhang, X.; Wu, X.; Jiang, Q.; et al. High-fat diet promotes liver tumorigenesis via palmitoylation and activation of AKT. *Gut* **2024**, *73*, 1156–1168, https://doi.org/10.1136/gutjnl-2023-330826.
17. Wang, J.; Hu, J.; Hu, H.; Guan, Q.; Zhu, Z.; Yang, Q.; Ding, G. APT1-derived depalmitoylation of CD36 alleviates diabetes-induced lipotoxicity in podocytes. *Int. J. Biol. Sci.* **2025**, *21*, 3852–3866, https://doi.org/10.7150/ijbs.109220.
18. Teran, E.; Tian, T.; Wang, C.; Wang, X.; Wang, J.; Ni, D.; Rathinam, V.A.; Ruan, J. Structural basis and regulation of GSDME pore formation. *Nat. Commun.* **2026**, *17*, https://doi.org/10.1038/s41467-026-70643-5.
19. Lakkaraju, A.K.; Abrami, L.; Lemmin, T.; Blaskovic, S.; Kunz, B.; Kihara, A.; Peraro, M.D.; van der Goot, F.G. Palmitoylated calnexin is a key component of the ribosome–translocon complex. *EMBO J.* **2012**, *31*, 1823–1835, https://doi.org/10.1038/emboj.2012.15.
20. Abrar, F.; Davies, M.C.; Alshehabi, Y.; Kumar, A.; Dang, A.; Nguyen, Y.T.N.; Collins, J.; Caron, N.S.; Choudhary, J.S.; Sanders, S.S.; et al. Reduced Palmitoylation of SQSTM1 /p62 in Huntington Disease Is Associated With Impaired Autophagy. *FASEB J.* **2025**, *39*, e70549, https://doi.org/10.1096/fj.202401781r.
21. Mukai, K.; Konno, H.; Akiba, T.; Uemura, T.; Waguri, S.; Kobayashi, T.; Barber, G.N.; Arai, H.; Taguchi, T. Activation of STING requires palmitoylation at the Golgi. *Nat. Commun.* **2016**, *7*, 11932, https://doi.org/10.1038/ncomms11932.
22. Lynes, E.M.; Bui, M.; Yap, M.C.; Benson, M.D.; Schneider, B.; Ellgaard, L.; Berthiaume, L.G.; Simmen, T. Palmitoylated TMX and calnexin target to the mitochondria-associated membrane. *EMBO J.* **2012**, *31*, 457–470, doi:10.1038/emboj.2011.384.
